# RNA-Binding Protein Signature in Proliferative Cardiomyocytes: A Cross-Species Meta-Analysis from Mouse, Pig, and Human Transcriptomic Profiling Data

**DOI:** 10.3390/biom15020310

**Published:** 2025-02-19

**Authors:** Thanh Nguyen, Kaili Hao, Yuji Nakada, Bijay Guragain, Peng Yao, Jianyi Zhang

**Affiliations:** 1 Department of Biomedical Engineering, School of Medicine and School of Engineering, University of Alabama at Birmingham, Birmingham, AL 35294, USA; thamnguy@uab.edu (T.N.); khao@uab.edu (K.H.); nkdyuji@uab.edu (Y.N.); bijayg@uab.edu (B.G.); 2 Aab Cardiovascular Research Institute, Department of Medicine, University of Rochester School of Medicine and Dentistry, Rochester, NY 14642, USA; 3 The Center for RNA Biology, University of Rochester School of Medicine and Dentistry, Rochester, NY 14642, USA; 4 Department of Biochemistry & Biophysics, University of Rochester School of Medicine and Dentistry, Rochester, NY 14642, USA; 5 Department of Medicine, Division of Cardiovascular Disease, School of Medicine, University of Alabama at Birmingham, Birmingham, AL 35294, USA

**Keywords:** RNA-binding protein, cardiomyocyte, cell cycle, heart regeneration

## Abstract

In mammals, because cardiomyocytes withdraw from cell-cycle activities shortly after birth, the heart cannot repair the damage caused by a myocardial injury; thus, understanding how cardiomyocytes proliferate is among the most important topics in cardiovascular sciences. In newborn neonatal mammals, when a left ventricular injury is applied in hearts earlier than postnatal day 7, the cardiomyocytes actively proliferate and regenerate lost myocardium in the following weeks. The regulators promoting cardiomyocyte proliferation were discovered by analyzing transcriptomic data generated from models. Most of these regulators support the mRNA production of cell-cycle machinery, yet the mRNA requires translation into functional proteins under the regulation of RNA-binding proteins (RBPs). In this work, we performed a meta-analysis to study the relationship between RBP expression and cardiomyocyte proliferation. To identify RBPs associated with mouse and pig cardiomyocyte proliferation, the single-nuclei RNA sequencing (snRNA-seq) data from regenerating mouse and pig hearts were reanalyzed via an Autoencoder focusing on RBP expression. We also generated and analyzed new bulk RNA-seq from two human-induced pluripotent stem cell-derived (hiPSC) cardiomyocyte (hiPSC-CM) cell lines; the first cell line was harvested sixteen days after differentiation, when the cells still actively proliferated, and the second cell line was harvested one hundred and forty days after differentiation, when the cells ceased cell cycle activity. Then, the RBP associated with mouse, pig, and hiPSC-CM were compared across species. Twenty-one RBPs were found to be consistently upregulated, and six RBPs were downregulated in proliferating mouse, pig, and hiPSC-derived cardiomyocytes. Among upregulated RBPs across species, an immunofluorescence-based imaging analysis validated the significant increase in the proteins of DHX9, PTBP3, HNRNPUL1, and DDX6 in pig hearts with proliferating CMs. This meta-analysis in all species demonstrated a strong relationship between RBP expression and cardiomyocyte proliferation.

## 1. Introduction

Despite ongoing improvements in the management of cardiac disease, patients with severe acute myocardial infarction (MI) often progress to end-stage congestive heart failure, which remains one of the most significant problems in public health. From the molecular and cellular perspectives, heart failure is caused by the loss of cardiomyocytes—the fundamental contractile units of the heart. Because mammalian cardiomyocytes exit the cell cycle shortly after birth, cardiomyocytes in the hearts of adult mammals cannot proliferate in response to injury. However, it was recently demonstrated that when MI was induced in the hearts of newborn mammals on less than postnatal day 7 (P7), the hearts could recover with a bit of residual fibrosis in the myocardium, and the repair process was accompanied by increases in the expression of markers for cell-cycle activity in the cardiomyocytes [1,2,3,4]. Therefore, deciphering how cardiomyocytes proliferate at the fetal-to-neonatal stage and exit the cell cycle at the neonatal-to-postnatal stage is among the most important topics in cardiovascular research [5,6,7]. To answer this question, in vitro and in vivo models have been generated in which cardiomyocytes actively proliferate. In vitro, after human-induced pluripotent stem cells (hiPSCs) were differentiated into cardiomyocyte linage (hiPSC-CM), the hiPSC-CM still actively proliferated approximately 2 weeks after differentiation [8,9,10]; then, three months after differentiation, the hiPSC-CMs ceased proliferating. In vivo, when myocardial infarction (MI) or apical resection (AR) was experimentally induced in pig and mouse hearts on postnatal day (P) 1, the animals recovered with no evidence of myocardial scarring or decline in contractile performance [1,3,11,12,13]. Furthermore, AR surgery on P1 prolonged the time window for myocardial repair for at least four weeks. Pigs that underwent both AR on P1 (AR_P1_) and MI on P28 (MI_P28_) completely regenerated the myocardial tissue that was damaged by the second injury [2], and the immunohistochemical expression of markers for cell-cycle activity and proliferation indicated that an increase in cardiomyocyte proliferation accompanied the repair process. On the other hand, unlike pigs and mice, adult zebrafish (>1 year old) hearts can completely regenerate from myocardial attacks [14,15,16,17]. Then, next-generation RNA deep sequencing, including bulk RNA-sequencing (RNA-seq) and single-cell/nuclei RNA-sequencing (sc/snRNA-seq) data, were generated from these models to identify critical regulators promoting cardiomyocyte proliferation [2,4,11,18,19].

The results from analyzing RNA-sequencing data led to the discovery of regulators in cardiomyocyte proliferation. These include mitogen-associated protein kinase [MAPK], Hippo, cyclic AMP [cAMP], Janus kinase/signal transducers and activators of transcription [JAK-STAT], and rat sarcoma [RAS] signaling pathways [18], nebulin (NEB), the metabolic regulator pyruvate kinase M1/2 (PKM1 and PKM2) [2], T-box transcription factors 5 and 20 (TBX5 and TBX20) [4], Forkhead box M1 (FOXM1) [8], GATA-binding protein 4 (GATA4) [20], bone morphogenetic protein (BMP), and Notch signaling pathways [21]. Most of these regulators act at the transcriptional level, facilitating the mRNA production of cell-cycle machinery genes. On the other hand, to fully activate the cell-cycle machinery after mRNA production, the mRNA requires translation to generate functional proteins. This process requires the activity of RNA-binding proteins (RBPs). The RBPs with known RNA-binding domains are called ‘classical RBPs’ [22,23,24]; meanwhile, the RBPs that bind to RNA yet are unclear in the binding domain are called ‘non-classical RBPs’ [22,25,26]. However, which RBPs participate in cardiomyocyte proliferation and what are their roles and mechanisms are still open questions [27].

In this work, we performed a meta-analysis to study the relationship between RBP expression and cardiomyocyte proliferation. The single-nuclei RNA-sequencing (snRNA-seq) data from mouse and pig hearts, in which cardiomyocytes actively proliferated, were reanalyzed via an Autoencoder focusing on RBP expression. In our previous analysis, when the Autoencoder technique was customized to focus only on cell-cycle gene expression, the ‘proliferating cardiomyocyte’ cluster was consistently identified in both mice [11] and pigs [2] and was defined as a cardiomyocyte cluster that co-upregulated five proliferation markers (AURKB, MKI67, INCENP, CDCA8, and BIRC5) [8]. We discovered that when the Autoencoder focuses only on RBP expression, the ‘proliferating cardiomyocyte’ could also be retrieved. We also generated a new bulk RNA-seq dataset from two hiPSC-CM cell lines: the first hiPSC-CM cell line was harvested sixteen days after differentiation (hiPSC-CM-D16), when the cell still actively proliferated, and the second hiPSC-CM cell line was harvested one hundred and forty days after differentiation (hiPSC-CM-D140), when the cells ceased cell-cycle activity. We demonstrate that the expression of many RBPs significantly differs between these two cell lines. An integrated analysis of transcriptomic profiling databases across three species indicates the presence of specific cohorts of RBPs as potential activators and repressors of cardiomyocyte proliferation shared by mice, pigs, and hiPSC-CMs, or one or two species among these three. This cross-species meta-analysis provides the scientific community with a valuable database to identify crucial RBPs for enhancing CM proliferation and heart regeneration.

## 2. Materials and Methods

### 2.1. snRNA-Seq Datasets

Two snRNA-seq datasets were analyzed via our RBP-specific analysis. The mouse datasets (GEO dataset GSE130699) [11] were collected from mouse hearts that underwent Sham and MI surgery on postnatal (P) day one and eight (Sham_P1_, MI_P1_, Sham_P8_, and MI_P8_, respectively), and hearts were explanted one (D1) or three (D3) days after the sham or MI induction procedure. The complete dataset contained data for 31,586 cells, including 25,065 cardiomyocytes. Pigs (GEO dataset GSE185289) [2] underwent apical resection surgery on P1 (AR_P1_), MI on P28 (MI_P28_), or both AR_P1_ and MI_P28_ (AR_P1_MI_P28_). The hearts of pigs that underwent MI_P1_ and AR_P1_MI_P28_ procedures were explained on P30, P35, P42, and P56. Also, the dataset included naïve (CTL) hearts explanted on P1, P28, and P56 and the hearts of fetal pigs on embryonic day (E) 80. The pig dataset had 250,700 cells, 129,991 of which were cardiomyocytes.

### 2.2. RBP-Specific Analyses in snRNA-Seq Data

snRNA-seq data integration, normalization, quality control, doublet filtering, and cell type identification were processed as described previously [2,8,28]. After separating the cardiomyocyte snRNA-seq data from data for other cell types, snRNA-seq data for the 6132 RBP genes [29] were extracted and processed via an Autoencoder. Using the same computational technique as our previous work [8], the Autoencoder embedded 6132 RBP gene expressions into 10 dimensions; then, the embedded data were visualized and clustered using the UMAP toolkit [30,31]. Upregulated genes were defined for each cluster via the following criteria: (1) cluster *p* < 10^−6^ (Wilcoxon rank-sum test [32]), (2) expression by at least 20% of cells in the cluster, and (3) mean abundance at least 2-fold greater for cells within the cluster than among cells in other clusters. The list of upregulated genes was analyzed with the DAVID functional annotation tool [33] to determine which pathways and biological processes were upregulated in each cluster; only terms present in the manually annotated Gene Ontology [34], KEGG [35], and Reactome [36] categories were selected, and to avoid false discoveries caused by multi-hypothesis testing, the results were required to have *p* < 0.01 or Benjamini-adjusted [37] *p* < 0.05. For each pathway and biological process, the enrichment score was calculated as the base-10 logarithm of the Benjamini-adjusted *p*-value.

### 2.3. Generating and Analyzing Bulk RNA-Seq Data from hiPSC-CM

The hiPSC-CM cell lines were generated as in our previous reports [8,38]. Briefly, human fibroblast cells were reprogrammed into hiPSCs; then, the hiPSC was differentiated into hiPSC-CMs. The cell lines were harvested at two different time points: sixteen days (hiPSC-CM-D16) and one hundred and forty days (hiPSC-CM-D140) after differentiation, with three culture plates (biological replicates) for each group. The hiPSC-CM-D16 cells (early stage) actively proliferated, while the hiPSC-CM-D140 cells did not, as previously reported [8,9,10]. The total RNAs were extracted from the cell lines as in our prior work [38]. The polyadenylated total RNAs were converted into complementary (c)DNA (reads) and then processed according to the Illumina NextSeq500 guidelines (https://support.illumina.com/downloads/nextseq-500-user-guide-15046563.html (accessed on 25 January 2025), San Diego, CA, USA). Then, the raw bulk RNA-seq data were generated and stored in a fastq file format.

The reads in fastq files were trimmed via trim-galore software [39]; then, via the STAR v.2.5 toolkit [40], the trimmed reads were mapped into the Human GRCh38 Reference Genome [41] to identify the gene corresponding to each read. The percentage of reads that can uniquely map to one gene in the Human Reference Genome was above 85% (Supplemental Table S1), indicating high data quality. Next, for each gene, the number of reads corresponding to the gene was counted via the HTSeq/0.6.1 package [42], which became the initial (non-normalized) gene expression in each replicate. Then, the initial gene expression was normalized, and the Deseq2 toolkit completed the statistical comparison between hiPSC-CM-D16 and hiPSC-CM-D140 cells [43]. The statistical *p*-values, computed via Deseq2, were adjusted using the Benjamini [37] method to resolve the false-discovery issue.

Upregulated genes in the hiPSC-CM-D16 cells were selected if the genes met the following criteria: (1) have an expression abundance (base mean, calculated by Deseq2 toolkit) of 100 or above, (2) have a mean abundance at least 2-fold greater compared to the hiPSC-CM-D140 cells, and (3) have Benjamini-adjusted *p*-values of 0.05 or less. Downregulated genes in the hiPSC-CM-D16 cells were identified using the same criteria. These genes were further analyzed by the DAVID functional annotation tool [33] to determine which pathways and biological processes were enriched in each cell group. The enrichment score was calculated as the base-10 logarithm of the Benjamini-adjusted *p*-value for each pathway and biological process.

### 2.4. Combining RBPs Associated with Cardiomyocyte Proliferation in Mouse, Pig, and hiPSC-CM Data

The entire list of RBPs was acquired from the RBP2GO database [29]. In mice and pigs, after clustering cardiomyocytes from snRNA-seq data via the RBP-specific analysis, each cluster was examined for either (1) the ‘proliferating’ CM cluster, in which all five proliferation markers (MKI67, AURKB, INCENP, BIRC5, and CDCA8) were co-upregulated, or (2) the ‘proliferation-associated’ CM cluster, with clusters that were enriched in hearts where cardiomyocyte proliferation was known to be active: Sham_P1_D1, MI_P1_D1, Sham_P1_D3, and MI_P1_D3 mouse hearts, and fetal, AR_P1_MI_P28_P30, AR_P1_MI_P28_P35, and AR_P1_MI_P28_P42 pig hearts. To determine whether a cardiomyocyte cluster is ‘proliferation-associated’, for each heart, the percentage (proportion) of cardiomyocytes belonging to the cluster was counted; then, a ‘proliferation-associated’ cluster was a cluster such that its proportion of Sham_P1_D1, MI_P1_D1, Sham_P1_D3, and MI_P1_D3 mouse hearts, as well as Fetal, AR_P1_MI_P28_P30, AR_P1_MI_P28_P35, and AR_P1_MI_P28_P42 pig hearts, was more than 2-fold higher than in the other group. These criteria were the same as in our previous work [8]. Upregulated genes in each ‘proliferating’ CM and ‘proliferation-associated’ CM were cross-checked in the RBP2GO database [29] to identify upregulated RBPs; then, the upregulated RBPs in all mouse (or pig) ‘proliferating’ CM and ‘proliferation-associated’ CM clusters were combined, resulting in an RBP list associated with cardiomyocyte proliferation. In hiPSC-CMs, upregulated genes in hiPSC-CM-D16 cells were also cross-checked in the RBP2GO database [29] to identify upregulated RBPs in proliferating hiPSC-CMs. The RPBs associated with proliferating mice, pigs, and hiPSC-CMs were analyzed for overlapping genes among two or three species.

### 2.5. Immunohistochemistry

An immunohistochemistry analysis was performed, as in our previous work [8], in which we demonstrated mitotic cardiomyocytes via phosphohistone H3 (PH3) staining. Frozen tissues were embedded in an optimal cutting temperature compound (Fisher Scientific; Waltham, MA, USA); cut into 10 μm sections, washed with PBS + 0.1% Tween 20 (PBST) for 10 min; fixed with 4% paraformaldehyde for 10 min at room temperature; permeabilized with chilled acetone for 3 min; washed with PBST; blocked with 10% donkey serum; incubated with fluorescently labeled DHX9 (Proteintech, 17721-1-AP, dilution 1:1000; Rosemont, IL, USA), PTBP3 (Invitrogen, PA598125, dilution 1:100; Carlsbad, CA, USA), HNRNPUL1 (Proteintech, 10578-1-AP, dilution 1:500; Rosemont, IL, USA), DDX6 (Santa Cruz, sc-376433, dilution 1:100; Dallas, TX, USA), RBMS3 (Invitrogen, PA5-114400, dilution 1:200; Carlsbad, CA, USA), or BICC1 (Santa Cruz, sc-514846, dilution 1:100; Dallas, TX, USA) antibodies overnight at 4 °C; washed 3 times with PBST; incubated with cardiomyocyte-specific marker antibodies, including fluorescently labeled cardiac troponin (cTnT) (Thermo Fisher, MA5-12960; Waltham, MA, USA) or α-Sarcomeric Actin (Sigma-Aldrich, A2172-100UL) antibody, and wheat germ agglutinin (WGA, Thermo Fisher, W32466) for 30 min; washed; mounted with antifade mounting medium containing 4′,6-diamidino-2-phenylindole (DAPI; Vector Laboratories; Newark, CA, USA); and imaged with an Olympus (Center Valley, PA, USA) FV3000 microscope. Three sections were evaluated per heart; 10 images were taken per section for gene expression analysis, and 20 images were taken per section to analyze the cardiomyocyte cross-sectional surface area and cardiomyocyte density. Actin or cTnT area was quantified with an in-house Matlab image segmentation program [8,44], and cardiomyocyte DAPI and nuclei-localized proteins (DHX9, PTBP3, and HNRNPUL1) were counted manually. For cytosol-localized proteins (DDX6 and BICC1), quantification was completed via light intensity on the Actin or cTnT area.

## 3. Results

### 3.1. RBP-Specific Analysis Re-Identified One ‘Proliferating’ CM Cluster and Three ‘Proliferation-Associated’ CM Clusters in Regenerative Mouse Hearts

**Figure 1:**
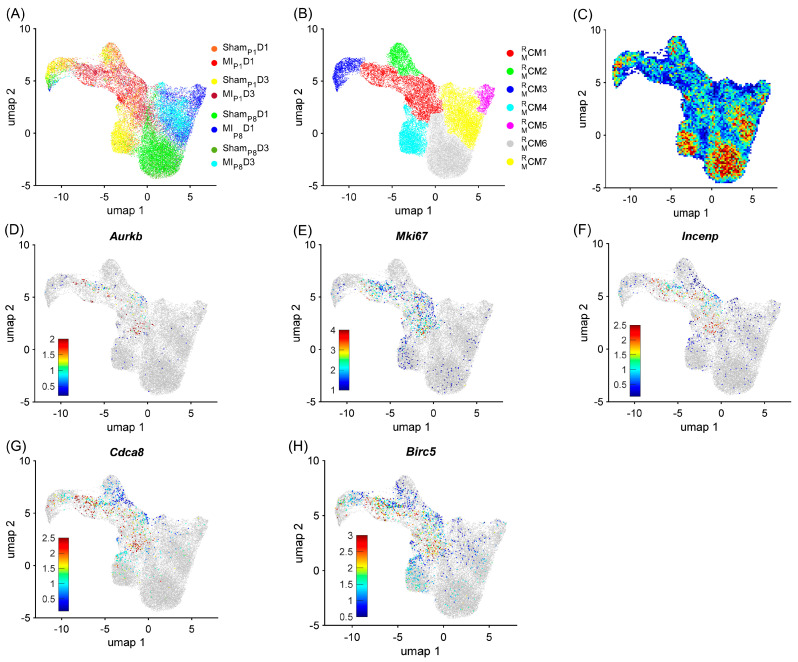
RBP-specific analysis identified seven mouse cardiomyocyte clusters (CM1MR−CM7MR). Mouse cardiomyocyte snRNA-seq data (8 animals, *n* = 25,065 cells) were processed via the RBP-specific Autoencoder and visualized in 2 dimensions (2D) via UMAP. (**A**) The cells were displayed and colored based on the injury and time point. (**B**) CM cluster locations and boundaries are identified in the 2D map. (**C**) The cell density in the 2D map, which indicated cluster tendency, was represented via a heatmap; areas with a high density of cells (cluster) were annotated in red. (**D**–**H**) The levels of expression for the proliferative markers (**D**) *Aurkb*, (**E**) *Mki67*, (**F**) *Incenp*, (**G**) *Cdca8*, and (**H**) *Birc5* in each cardiomyocyte are displayed as a heatmap; gray cardiomyocytes did not express the corresponding marker.

**Figure 2:**
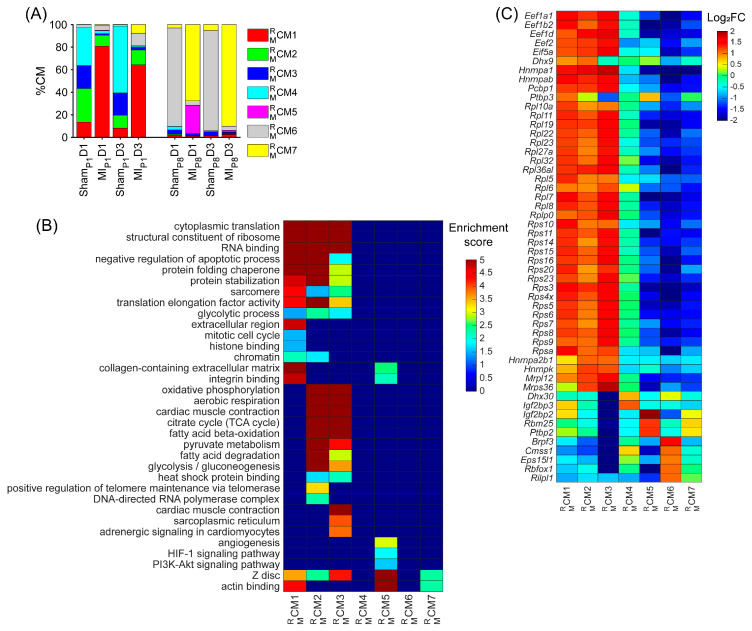
Upregulated RBP genes, enriched pathways, and biological process of each mouse cardiomyocyte cluster. (**A**) The proportion of cardiomyocytes from each cluster is displayed for each injury group and time point. (**B**) For each cluster, the enrichment score for each pathway and biological process is displayed in a heatmap. (**C**) The abundance of expression (normalized via z-score) for classical RBP genes in each cluster is displayed as a heatmap.

To demonstrate the strong relationship between the RBP and the cell-cycle machinery in mice, cardiomyocytes were re-clustered via the RBP-specific Autoencoder, in which only RNA expression (from snRNAseq data) of RBP genes was analyzed. We hypothesize that if the RBP and the cell-cycle machinery have a strong relationship, the RBP-specific Autoencoder (without cell-cycle-specific data) would retrieve the cell-cycle cardiomyocyte cluster. The overall two-dimensional (2D) landscape of cardiomyocytes in each mouse heart showed that the batch effect was negligible in an RBP-specific analysis because no major cardiomyocyte clusters were exclusive for a single heart group (Figure 1A and Figure S1A–H). The RBP-specific analysis found seven mouse cardiomyocyte clusters (CM1MR−CM7MR, respectively). All five proliferation markers (*Mki67*, *Aurkb*, *Incenp*, *Cdca8*, and *Birc5*) were co-enriched in the CM1MR cells (Figure 1B–G, Supplemental Table S2). Furthermore, CM1MR was the major cardiomyocyte cluster (>60%) in MI_P1_D1 and MI_P1_D3 hearts; CM1MR comprised 13.51% and 8.14% of Sham_P1_D1 and Sham_P1_D3 hearts, respectively; and the CM1MR proportion was very low (<2.5%) in other mouse hearts where cardiomyocytes did not actively proliferate (Figure 2A and Figure S1I). Also, the mitotic cell cycle, chromatin, and histone-binding processes were enriched in CM1MR (Figure 2B, Supplemental Table S3). Altogether, these observations suggested that CM1MR was the only cluster containing ‘proliferating’ cardiomyocytes in mouse snRNA-seq data. On the other hand, the negative regulator of the apoptotic process was also enriched in CM1MR, which suggested that cells not only activated the cell cycle but also protective mechanisms, especially under MI_P1_ injury.

Besides the cell-cycle cardiomyocyte cluster CM1MR, we assessed whether other clusters could be associated with the proliferative phenotype. The cluster CM2MR was only presented in Sham_P1_D1, MI_P1_D1, Sham_P1_D3, and MI_P1_D3 hearts (Figure 2A and Figure S1J); here, positive regulators of telomere maintenance, which is known to be critical in the cell-cycle process [45], were enriched (Figure 2B, Supplemental Table S3). The cluster CM3MR comprised approximately 20% Sham_P1_D1, and Sham_P1_D3 hearts; meanwhile, the CM3MR proportions were less than 5% in other groups (Figure 2A and Figure S1K). Regulators of glycolysis, β-fatty acid oxidation, oxidative phosphorylation, and citrate cycle were enriched in both CM2MR and CM3MR (Figure 2B, Supplemental Table S3). Also, the cluster proportions CM4MR were explicitly high in Sham_P1_D1 and Sham_P1_D3 hearts (Figure 2A and Figure S1L). Thus, clusters CM2MR, CM3MR, and CM4MR were determined as ‘proliferation-associated’ CM clusters. Otherwise, clusters CM5MR, CM6MR, and CM7MR were the major cardiomyocyte clusters in Sham_P8_D1, MI_P8_D1, Sham_P8_D3, and MI_P8_D3 hearts (Figure 2A and Figure S1M–O); therefore, these clusters were unlikely to be associated with cardiomyocyte cell-cycle activities.

Being specific for post-transcriptional processes, we found that the regulators of RNA binding, the structure constituent of ribosomes, protein-folding chaperones, and translation elongation factors were significantly enriched throughout clusters CM1MR, CM2MR, and CM3MR (Figure 2B, Supplemental Table S3). More specifically, the expressions of the well-known eukaryotic translation elongation factor 1 subunits alpha 1, beta 2, and delta ([*Eef1a1*], [*Eef1b2*], and [*Eef1d*], respectively); eukaryotic translation elongation factor 2 [*Eef2*]; eukaryotic translation initiation factor 5A [*Eif5a*; a translation elongation factor]; and many ribosomal proteins (12 large subunit proteins [*Rpl*] and 15 small subunit proteins [*Rps*]) were especially high in the CM1MR, CM2MR, and CM3MR clusters and were medium in the CM4MR cluster but were low in the CM5MR, CM6MR, and CM7MR clusters (Figure 2C). Together, there were 258 upregulated RBPs in the mouse ‘proliferating’ CM and ‘proliferation-associated’ CM clusters (Table S1A), including 52 classical RBPs.

### 3.2. RBP-Specific Analysis Re-Identified Two ‘Proliferating’ CM Clusters and Two ‘Proliferation-Associated’ CM Clusters in Pig Hearts

**Figure 3:**
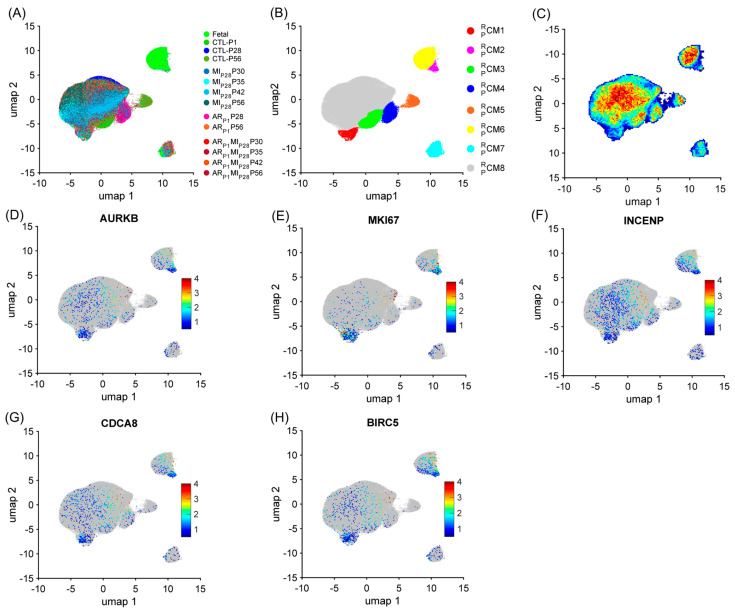
The RBP-specific analysis identified eight pig cardiomyocyte clusters (CM1PR−CM8PR). Pig cardiomyocyte snRNA-seq data (38 animals, *n* = 129,991 cells) were processed via the RBP-specific Autoencoder and visualized in 2D via UMAP. (**A**) The cells were displayed and colored based on the animal, injury, and time point. (**B**) CM cluster locations and boundaries are identified in the 2D map. (**C**) The cell density in the 2D map, which indicated cluster tendency, was represented via a heatmap; areas with a high density of cells (cluster) were annotated in red. (**D**–**H**) The levels of expression for the proliferative markers (**C**) AURKB, (**D**) MKI67, (**E**) INCENP, (**F**) CDCA8, and (**G**) BIRC5 in each cardiomyocyte are displayed as a heatmap; gray cardiomyocytes did not express the corresponding marker.

**Figure 4:**
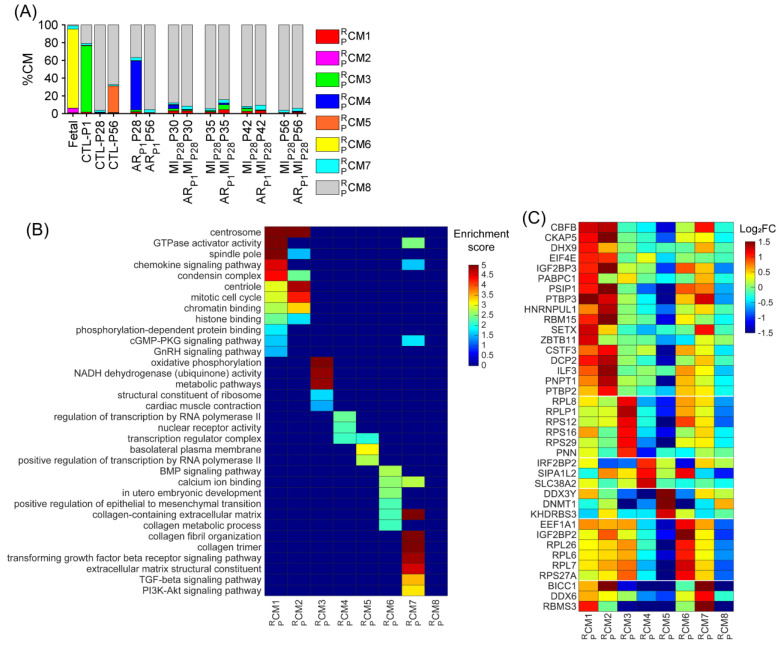
Upregulated RBP genes, enriched pathways, and biological process of each pig cardiomyocyte cluster. (**A**) The proportion of cardiomyocytes from each cluster is displayed for each injury group and time point. (**B**) For each cluster, the enrichment score for each pathway and biological process is displayed in a heatmap. (**C**) The abundance of expression (normalized via z-score) for classical RBP genes in each cluster is displayed as a heatmap.

Similar to the mouse data analysis, cardiomyocytes were re-clustered via the RBP-specific Autoencoder to demonstrate the strong relationship between the RBP and the cell-cycle machinery in pigs. The overall 2D landscape of cardiomyocytes in each pig heart group showed the fetal cardiomyocytes were separated from the cardiomyocytes from other groups, which was consistent with our previous reports [2,4,28]. Otherwise, because no major and separated pig cardiomyocyte clusters were exclusive for a single heart group, the batch effect was negligible in the RBP-specific analysis (Figure 3A and Figure S2A–N). The RBP-specific analysis found eight mouse cardiomyocyte clusters (CM1PR−CM8PR, respectively). All five proliferation markers (*MKI67*, *AURKB*, *INCENP*, *CDCA8*, and *BIRC5*) were co-enriched in the CM1PR and CM2PR clusters (Figure 3B–G, Supplemental Table S4). The proportion of CM1PR was low (<2%) in all groups, except in AR_P1_MI_P28_P30 (4.38%) and AR_P1_MI_P28_P35 (3.48%) hearts (Figure 4A and Figure S2O); meanwhile, CM2PR was only presented in the fetal hearts (Figure 4A and Figure S2P). Furthermore, the mitotic cell cycle, centrosomes, chromatin binding, and the condensin complex were enriched in both CM1PR and CM2PR (Figure 4B, Supplemental Table S5). Altogether, these observations suggested that CM1PR and CM2PR were the ‘proliferating’ CM clusters in pig snRNA-seq data.

Besides the retrieved cluster CM1MP, we assessed whether other pig cardiomyocyte clusters could be associated with the proliferative phenotype. The cluster CM3PR and also the major cardiomyocyte cluster (74.61%) were only presented for CTL-P1 pig hearts; the cluster CM4PR was exclusive for AR_P1_P28 pig hearts (55.21%); the cluster CM5PR was exclusive for CTL-P56 hearts; and cluster CM6PR was exclusive for fetal hearts (Figure 4A and Figure S2Q–T). The cluster CM7PR comprised 3.79% fetal cardiomyocytes; then, the percentage of CM7PR gradually decreased in postnatal naïve hearts toward 1.90% in CTL-P28 and 1.36% in CTL-P56 cardiomyocytes. Following AR_P1_ injury, the percentage of CM7PR remained at 3.38% in AR_P1_P28 and 3.22% in AR_P1_P56 hearts. In MI_P28_-only hearts, CM7PR percentages did not exceed 2.1%; however, in AR_P1_MI_P28_ hearts, CM7PR percentages elevated toward 3.5% on P30, then 3.82% on P35, and 5.28% on P42, before decreasing toward 3.18% on P56 (Figure 4A and Figure S2U). Also, the transforming growth factor beta (TGFβ) and PI3K-AKT signaling pathways, which promote cardiomyocyte proliferation [46,47], were enriched in CM7PR (Figure 4B, Supplemental Table S5). Meanwhile, cluster CM8PR was the major cardiomyocyte cluster (>60%) in CTL-P28, CTL-P56, AR_P1_-P56, as well as MI_P28_-only and AR_P1_MI_P28_ hearts. Altogether, CM6PR and CM7PR were two ‘proliferation-associated’ CM clusters in pig snRNA-seq data.

Specifically, for RBP, combining the upregulated RBPs in the CM1PR, CM2PR, CM6PR, and CM7PR clusters resulted in 317 genes (Table S1A), including 38 classical RBPs (Figure 4C). Genes that were highly upregulated in the CM1PR and CM2PR clusters were also highly expressed in the CM6PR and CM7PR clusters (compared to CM4PR, CM5PR, and CM8PR), such as core-binding factor subunit beta [*CBFB*], DExH-box helicase 9 [*DHX9*], insulin-like growth factor 2 mRNA-binding protein 3 [*IGF2BP3*], eukaryotic translation initiation factor 4E [*EIF4E*], poly(A)-binding protein cytoplasmic 1 [*PABPC1*], PC4 and SRSF1 interacting protein 1 [*PSIP1*], polypyrimidine tract-binding protein 3 [*PTBP3*], and heterogeneous nuclear ribonucleoprotein U like 1 [*HNRNPUL1*] (Figure 4C). Genes of ribosomal proteins were upregulated in the CM1PR, CM2PR, CM3PR, CM6PR, and CM7PR clusters.

### 3.3. Differentially Expressed RBPs in hiPSC-CM Bulk RNA-Seq Analysis

**Figure 5:**
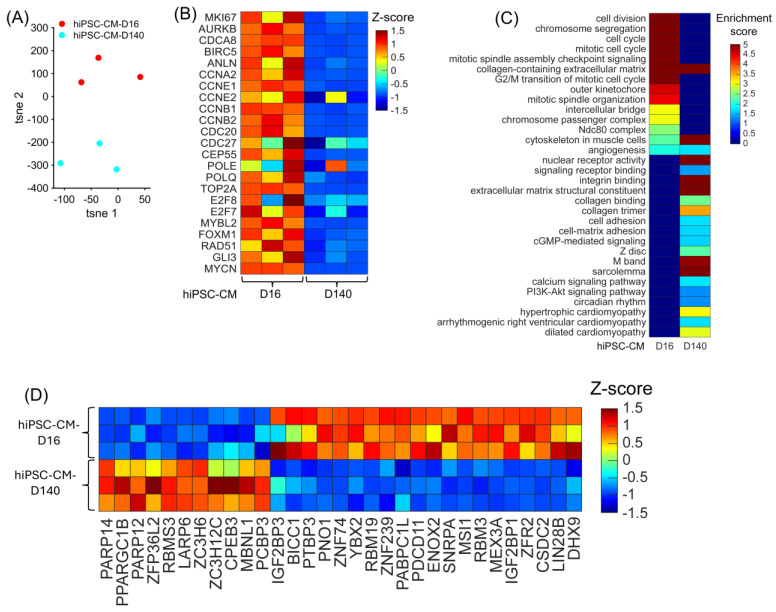
Upregulated RBP genes, enriched pathways, and biological process when comparing hiPSC-CM-D16 with hiPSC-CM-D140 cell lines. (**A**) The expression of all genes in each cell line replicates are embedded and displayed in a 2D UMAP; each dot represents a biological replicate, and the color indicates the cell line group. (**B**) The abundance of expression for cell-cycle regulators in each cell line replicate is displayed as a heatmap. (**C**) For each cell line, the enrichment score for each pathway and biological process is displayed in a heatmap. (**D**) The abundance of expression (normalized via z-score) for classical RBP genes in each replicate is displayed as a heatmap.

To determine whether the bulk RNA-seq data reflected the phenotypic difference between the hiPSC-CM-D16 and the hiPSC-CM-D140 cells, the data from all replicates were visualized in a 2D plot, in which separations between hiPSC-CM-D16 and hiPSC-CM-D140 replicates were expected. Also, expressions of cell-cycle markers between the two cell lines were compared and visualized in a heatmap. The t-distributed stochastic neighbor embedding (tSNE) plot [48], in which the expression of all genes in the bulk RNA-seq data was embedded into just two dimensions, showed a clear separation between the hiPSC-CM-D16 and hiPSC-CM-D140 replicates (Figure 5A). All five proliferation markers (*MKI67*, *AURKB*, *INCENP*, *CDCA8*, and *BIRC5*) and other critical cell-cycle regulators were significantly upregulated in the hiPSC-CM-D16 cell line (Figure 5B, Supplemental Table S6), including Cyclin E1 [*CCNE1*], Cyclin E2 [*CCNE2*], Cyclin B1 [*CCNB1*], Cyclin B2 [*CCNB2*], MYB proto-oncogene like 2 [*MYBL2*], Forkhead box M1 [*FOXM1*], GLI family zinc finger 3 [*GLI3*], E2F transcription factor 8 [*E2F8*], and RAD51 recombinase [*RAD51*]. Furthermore, cell-cycle processes were significantly enriched in the hiPSC-CM-D16 cell line; meanwhile, genes constituting the Z disc, M band, sarcolemma, and cytoskeleton in muscle cells were significantly enriched in the hiPSC-CM-D140 cell line, consistent with a more mature and non-proliferating state (Figure 5C, Supplemental Table S7). Thus, the bulk RNA-seq data strongly represents the proliferative phenotype of the hiPSC-CM cell early (16 days) after differentiation. A total of 2591 genes were differentially expressed between hiPSC-CM-D16 and the hiPSC-CM-D140 cells, including 754 RBPs (Supplemental Table S6). Among them, 419 RBPs were upregulated in the hiPSC-CM-D16 cells, including 21 classical RBPs (Figure 5D).

### 3.4. Overlapping CM-Proliferation-Associated RBP Among Mice, Pig, and hiPSC-CM Transcriptomic Profiling Data

**Figure 6:**
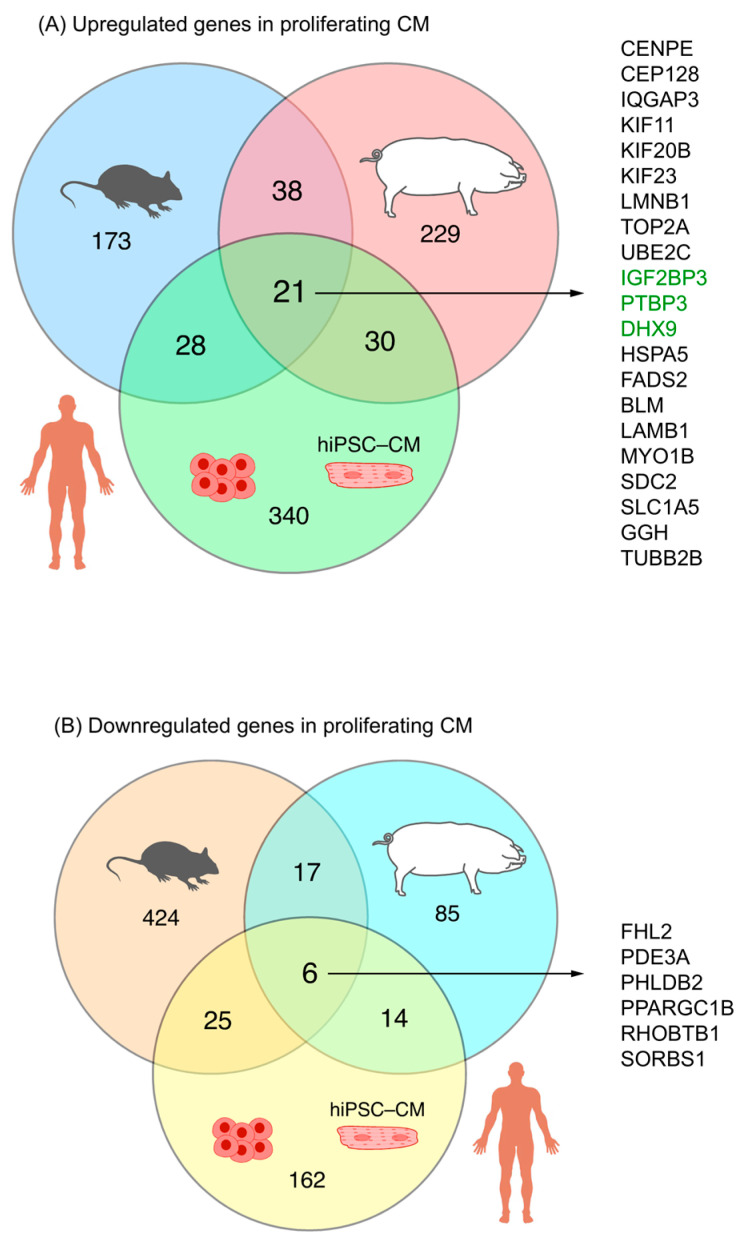
The overlap among RBPs associated with cardiomyocyte proliferation across mice, pigs, and hiPSC-CMs is displayed in a Venn diagram. (**A**) Upregulation in mouse and pig ‘proliferating’/‘proliferation-associated’ CM clusters, and hiPSC-CM-D16. Classical RBPs are highlighted in green. (**B**) Downregulation in mouse and pig ‘proliferating’/‘proliferation-associated’ CM clusters, and hiPSC-CM-D16.

Overall, twenty-one RBPs were associated with cardiomyocyte proliferation in mice, pigs, and hiPSC-CMs (Figure 6A). They were Bloom syndrome RecQ like helicase [*BLM*], centromere protein E [*CENPE*], centrosomal protein 128 [*CEP128*], DExH-box helicase 9 [*DHX9*], fatty acid desaturase 2 [*FADS2*], gamma-glutamyl hydrolase [*GGH*], heat shock protein family A (Hsp70) member 5 [*HSPA5*], insulin-like growth factor 2 mRNA-binding protein 3 [*IGF2BP3*], kinesin family member 11 [*KIF11*], kinesin family member 20B [*KIF20B*], kinesin family member 23 [*KIF23*], lamin B1 [*LMNB1*], laminin subunit beta 1 [*LAMB1*], myosin IB [*MYO1B*], polypyrimidine tract-binding protein 3 [*PTBP3*], syndecan 2 [*SDC2*], solute carrier family 1 member 5 [*SLC1A5*], DNA topoisomerase II alpha [*TOP2A*], IQ motif containing GTPase activating protein 3 [*IQGAP3*], tubulin beta 2B class IIb [*TUBB2B*], and ubiquitin-conjugating enzyme E2 C [*UBE2C*] (Supplemental Figure S3). Otherwise, 37 RBPs were upregulated in the ‘proliferating’ and ‘proliferation associated’ CM clusters in both pigs and mice but were not upregulated in the hiPSC-CM-D16 cells (Supplemental Table S8). There were 30 RBPs associated with cardiomyocyte proliferation in pigs and hiPSC-CMs but not found to be associated with proliferating mouse cardiomyocytes (Supplemental Table S8). Also, 28 RBPs were associated with cardiomyocyte proliferation in mice and hiPSC-CMs, but they were not found to be associated with proliferating pig cardiomyocytes (Supplemental Table S8). On the other hand, overlapping RBPs that were downregulated in proliferating mouse and pig cardiomyocyte clusters but upregulated in hiPSC-CM-D16 yielded six genes (Figure 6B, Tables S1B and S9): sorbin and SH3 domain containing 1 [*SORBS1*], peroxisome proliferator-activated receptor gamma, coactivator 1 beta [*PPARGC1B*], Rho related BTB domain containing 1 [*RHOBTB1*], pleckstrin homology like domain family B member 2 [*PHLDB2*], phosphodiesterase 3A [*PDE3A*], and four and a half LIM domains 2 [*FHL2*].

### 3.5. Immunohistochemistry Analysis of Upregulated Classical RBPs at the Proliferative Stages of Cardiomyocytes in Pig Hearts

**Figure 7:**
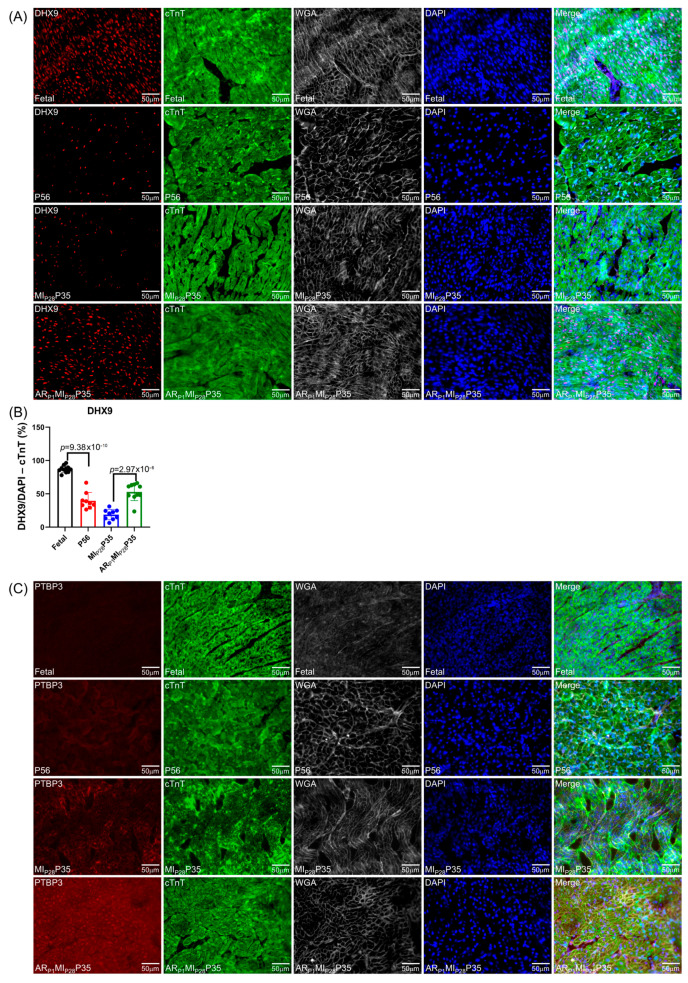

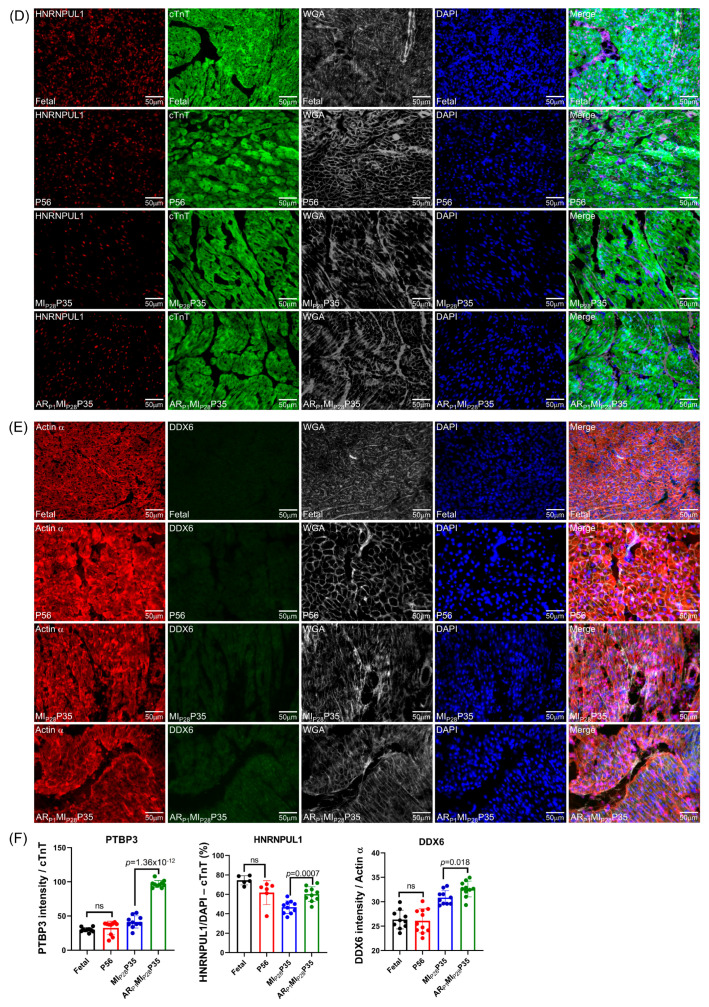

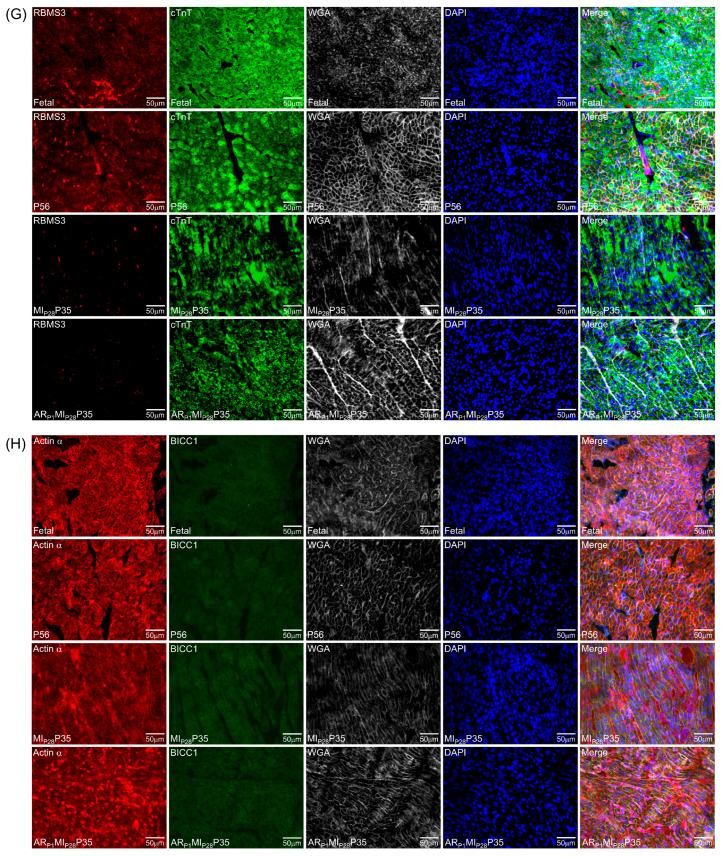
Immunohistochemistry analysis of upregulated classical RBPs in pig cardiomyocyte. (**A**,**C**–**G**) Representative images; sections from fetal and CTL-P56 hearts and the left-ventricle border zone of MI_P28_P35 and AR_P1_MI_P28_P35 were stained for the presence of DHX9 ((**A**), red), PTBP3 ((**C**), red), HNRNPUL1 ((**D**), red), DDX6 ((**E**), green), RBMS3 ((**G**), red), and BICC1 ((**H**), green); then, cTnT ((**A**,**C**,**D**,**G**) green) or Actin α ((**E**,**H**), red) and with WGA (white) to identify cell borders; nuclei were labeled with DAPI (blue). (**B**) The percentage of cardiomyocytes expressing DDX9 (**B**) and HNRNPUL1 (**F**), as well as the intensity on cTnT (or Actin) of PTBP3 and DDX6 (**F**), were quantified and displayed in a bar graph. Each dot in panels (**B**,**F**) represents the results from a single image (n ≥ 10 per group). *p*-values were determined via Student’s *t*-test.

To further confirm the important RBPs identified in the RNA analysis (Figure 4C) at the protein level, we performed an immunofluorescence analysis of multiple RBP expressions in regenerative and non-regenerative pig hearts. We observed a significant increase in the protein expression levels of DHX9 in fetal and AR_P1_MI_P28_P35 pig cardiomyocytes compared to CTL-P56 and MI_P28_P35, respectively (Figure 7A). Moreover, the expressions of PTBP3, HNRNPUL1, and DDX6 were not significantly different between fetal and CTL-P56 cardiomyocytes; however, their expression increased in AR_P1_MI_P28_P35 cardiomyocytes compared to MI_P28_P35 ones (Figure 7B–D). However, we did not observe a significant difference in RBMS3, which appeared primarily expressed in non-cardiomyocytes, and BICC1 among these cardiomyocyte groups (Figure 7E).

## 4. Discussion

This report presents our fourth analysis of the same snRNA-seq data collected from fetal, neonatal, and postnatal pigs and our third analysis of the same snRNA-seq dataset from neonatal and postnatal mice [2,4,8,28]. In the latest analysis, when the Autoencoder was customized to focus only on the expression of cell-cycle genes, the ‘cycling cardiomyocyte’ cluster was identified by the co-upregulation of all five proliferation markers (*MKI67*, *AURKB*, *INCENP*, *CDCA8*, and *BIRC5*). In this analysis, when the Autoencoder only focused on the expression of RNA-binding proteins, cardiomyocyte clusters, in which all proliferation markers were co-upregulated, were also retrieved in both pig and mouse snRNA-seq data. Furthermore, a large number (614 genes) of RBPs were upregulated in the hiPSC-CMs on day sixteen after differentiation, when cells still actively proliferated. Together, the analysis of three different species suggested a strong relationship between RBP expression and cardiomyocyte proliferation.

Among twenty-one RBPs consistently found to be associated with cardiomyocyte proliferation in mouse, pig, and hiPSC-CM RNA-sequencing data, only IGF2BP3 has been reported to promote adult myocardial regeneration in mice [49]. CENPE and CEP128 are located in the centromere [50], centriole, and spindle pole [51]; TOP2A is a regulator in DNA replication and DNA damage repair [52]; and KIF11 and KIF20B regulate the spindle dynamics [53,54,55]. Thus, the role and mechanism of how TOP2A, CENPE, CEP128, FANCD2, KIF11, KIF20B, and KIF23 regulate cardiomyocyte proliferation could be similar to previous reports [50,51,53,54,55]. Therefore, whether and through which mechanism BLM, DHX9, FADS2, GGH, HSPA5, LAMB1, MYO1B, PTBP3, SDC2, SLC1A5, LMNB1, IQGAP3, TUBB2B, and UBE2C promote cardiomyocyte proliferation is yet to be studied. Among these twenty RBPs, we noticed nine of them are associated with ribosomes [56], including BLM, DHX9, HSPA5, IGF2BP3, KIF20B, LMNB1, MYO1B, TOP2A, and TUBB2B. This indicates their potential role in regulating mRNA translation despite some RBPs primarily localized in the nucleus and also in shuttling to the cytoplasm, such as DHX9 and PTBP3. Moreover, except DHX9, PTBP3, and IGF2BP3, eighteen of these upregulated genes in proliferating CMs encode nonclassical RBPs, such as metabolic enzymes, heat shock proteins, and cytoskeletal factors, among others, which warrant further investigation into how they influence gene regulation and mRNA metabolism in cardiomyocyte proliferation.

Many mitochondrial or cytoplasmic metabolic enzymes are categorized as nonclassical RBPs across different cell types, including 73 RBPs identified by proteomic mass spectrometry screening following mRNA interactome capture in mouse CMs [57]. We noticed increased expression of multiple metabolic enzymes involved in the redox signaling (e.g., MGST1 in proliferating pig neonatal CMs and hiPSC-CMs), electron transport chain (e.g., NDUFC2 and COX5B in proliferating mouse and pig neonatal CMs), fatty acid oxidation (e.g., ACADVL in proliferating mouse and pig neonatal CMs), mitochondrial RNA modification (e.g., PUS1 and LARS2 in proliferating hiPSC-CMs), and tricarboxylic acid cycle (e.g., IDH2, MDH2, and PDHA1 in proliferating mouse neonatal CMs) (Supplemental Figure S4, Supplemental Table S10). Some of these metabolic enzymes are well-known classical RBPs, such as PUS1 for modifying mRNA with pseudouridylation and LARS2 for catalyzing Leu-tRNA^Leu^ synthesis (Supplemental Figure S4). The other metabolic enzymes are novel nonclassical RBPs, such as IDH2, MDH2, and PDHA1 (Supplemental Table S10), confirmed as enabling RNA binding by UV-crosslinking and immunoprecipitation assays [57]. The function and mechanism of these moonlighting RNA-binding metabolic enzymes would be an intriguing question to address in future studies.

The mRNA expression is sometimes not associated with protein expression due to post-transcriptional regulation such as mRNA decay and translational control. We also examined the protein expression status in previously published whole-heart mass spectrometry-based proteomic databases to provide further supporting evidence. For two classic RBPs, DHX9 and PTBP3, protein expression is 2.04-fold (Log_2_FC = 1.03; *p_adj_* = 6.08 × 10^−3^) and 1.27-fold (Log_2_FC = 0.344; *p* = 0.12) higher at P1 than that at P28, respectively [58]. The protein expression of a large number of ribosome proteins is also significantly higher at P1 than at P28, such as RPL29 (Log_2_FC = 1.7; *p_adj_* = 3.40 × 10^−4^), RPL3 (Log_2_FC = 1.62; *p_adj_* = 2.74 × 10^−4^), and RPS27 (Log_2_FC = 1.82; *p_adj_* = 5.15 × 10^−4^), among many others. As a positive control, IGF2BP3 protein is expressed higher (Log_2_FC = 1.18; *p_adj_* = 0.01) at P1 than at P28 [11]. Moreover, DHX9 and IGF2BP3 protein expressions are even higher at P1 than at P56 (Log_2_FC = 1.72; *p_adj_* = 3.39 × 10^−4^ and Log_2_FC = 1.65; *p_adj_* = 1.73 × 10^−3^, respectively). However, PTBP3 protein level is slightly higher at P1 than at P56 (Log_2_FC = 0.306; *p* = 0.17). In contrast, the expression of all three proteins is unchanged at P1 compared to that at P7 within the proliferative time window (DHX9: Log_2_FC = 0.22; *p_adj_* = 0.52; IGF2BP3: Log_2_FC = 0.03; *p_adj_* = 0.95; PTBP3: Log_2_FC = 0.04; *p* = 0.83), suggesting that the protein expression of these RBPs may decline after P1–P7 and is reduced more at P56 than at P28. Intriguingly, we noticed that two pairs of other protein family members, IGF2BP1 and IGFBP2, as well as PTBP1 and PTBP2, showed significantly higher protein expressions at P1 than at P28 and P56 but remained unchanged at P7 versus P1. Considering the absence of these family members in our transcriptomic meta-analysis, these protein family members may play complementary or synergistic roles to IGF2BP3 and PTBP3, respectively. This is consistent with the high expression of *IGF2BP1*, *IGF2BP2*, and *IGF2BP3* at the mRNA level in neonatal mouse cardiomyocytes compared to postnatal ones [11]. Similarly, multiple other RNA helicases showed comparable protein expression patterns to DHX9, including DHX29, DHX30, DDX5, DDX60, DDX21, DDX39A, and DDX47. These proteomic data reveal the potential false-negative genes missed in our transcriptomic meta-analysis.

The discrepancy of classical RBP quantification between the transcriptomic (via snRNA-seq) and proteomic (via immunohistochemistry, Figure 7) highlights the necessity of examining the protein expression in addition to the mRNA expression, as they may not always correlate with each other. Also, we may not underestimate the importance of any candidate RBP revealed by snRNA-seq or bulk RNA-seq across two species (humans and pigs, humans and mice, and pigs and mice) or only within a single species (humans, pigs, or mice) to avoid missing potential false-negative hits that could play a role in cardiomyocyte proliferation and heart regeneration.

On the other hand, we examined six RBPs (CBX7, HAPLN1, LIN28A, MBNL1, METTL3, and MNK2) that were reported to promote cardiomyocyte proliferation between 2021 and 2024 [59,60,61,62,63,64,65] (Supplemental Table S11). None of these RBPs were presented in two or more RBP lists (mouse, pig, and hiPSC-CM) from our analysis. For example, LIN28A was only found in hiPSC-CM analyses, and CBX7 was only identified in pig snRNA-seq analyses. Also, Figure 6 shows that only a small proportion of the RBPs associated with cardiomyocyte proliferation overlapped among the three species. These findings suggest that the high heterogeneity in RBPs may contribute to regulating cardiomyocyte proliferation across different species.

## 5. Conclusions

In conclusion, we have created a new RBP-specific analytic method, reanalyzed the previously generated cardiomyocyte snRNA-seq data in regenerative hearts, and analyzed RBP gene expression in bulk RNA-seq data collected from proliferative hiPSC-CMs. The RBP-specific analysis retrieved the proliferative cardiomyocyte clusters with cell-cycle activities in all three species, and twenty-one RBPs were found to be consistently upregulated in proliferating mouse, pig, and human iPSC-derived cardiomyocytes.

## Data Availability

The bulk RNA sequencing data of hiPSC-CM cell lines is publicly available in the Gene Expression Omnibus database, accession number GSE289839 (https://www.ncbi.nlm.nih.gov/geo/query/acc.cgi?acc=GSE289839).

## Supplementary Materials

The following supporting information can be downloaded at: https://www.mdpi.com/article/10.3390/biom15020310/s1, Figure S1. The mouse cardiomyocyte data in Figure 1 and Figure 2 are enlarged for clarity. Figure S2. The pig cardiomyocyte data in Figure 3 and Figure 4 are enlarged for clarity. Figure S3. The abundance of expression of 21 RBPs, which is associated with cardiomyocyte proliferation in mouse, pig, and hiPSC-CM data, were displayed together. Figure S4. The overlap among mitochondrial proteins as nonclassical RBPs and associated with cardiomyocyte proliferation. Table S1. Statistics of the hiPSC-CM bulk RNA-seq data, when the RNA reads, were mapped into the Human GRCh38 reference genome. The statistics were produced by the STAR v.2.5.2 toolkit. Table S2. Statistics of upregulated genes in each mouse cardiomyocyte cluster. Table S3. Statistics of enriched pathways and biological processes in each mouse cardiomyocyte cluster. Table S4. Statistics of upregulated genes in each pig cardiomyocyte cluster. Table S5. Statistics of enriched pathways and biological processes in each pig cardiomyocyte cluster. Table S6. Statistics of differentially-expressed genes, compared between hiPSC-CM-D16 and hiPSC-CM-D14 cell lines. Table S7. Statistics of enriched pathways and biological processes in (A) hiPSC-CM-D16 and (B) hiPSC-CM-D140 cell lines. Table S8. Expression trend of each RBP in mouse, pig, and hiPSC-derived cardiomyocyte comparisons. Four column headers correspond to the trend in four comparisons. Table S9. Statistics of downregulated RBP genes in each mouse and pig cardiomyocyte cluster. Table S10. The list of mitochondrial proteins as nonclassical RBPs associated with cardiomyocyte proliferation in mouse, pig, and hiPSC-CM data. Table S11. Literature reviews of RBPs in heart, published between 2004 and 2024.
