# Supplementary figures and images for: RNA-Binding Protein Signature in Proliferative Cardiomyocytes: A Cross-Species Meta-Analysis from Mouse, Pig, and Human Transcriptomic Profiling Data
Source: Biomolecules. 2025 Feb 19;15(2):310. doi: 10.3390/biom15020310 (PMC11853426; doi:10.3390/biom15020310)

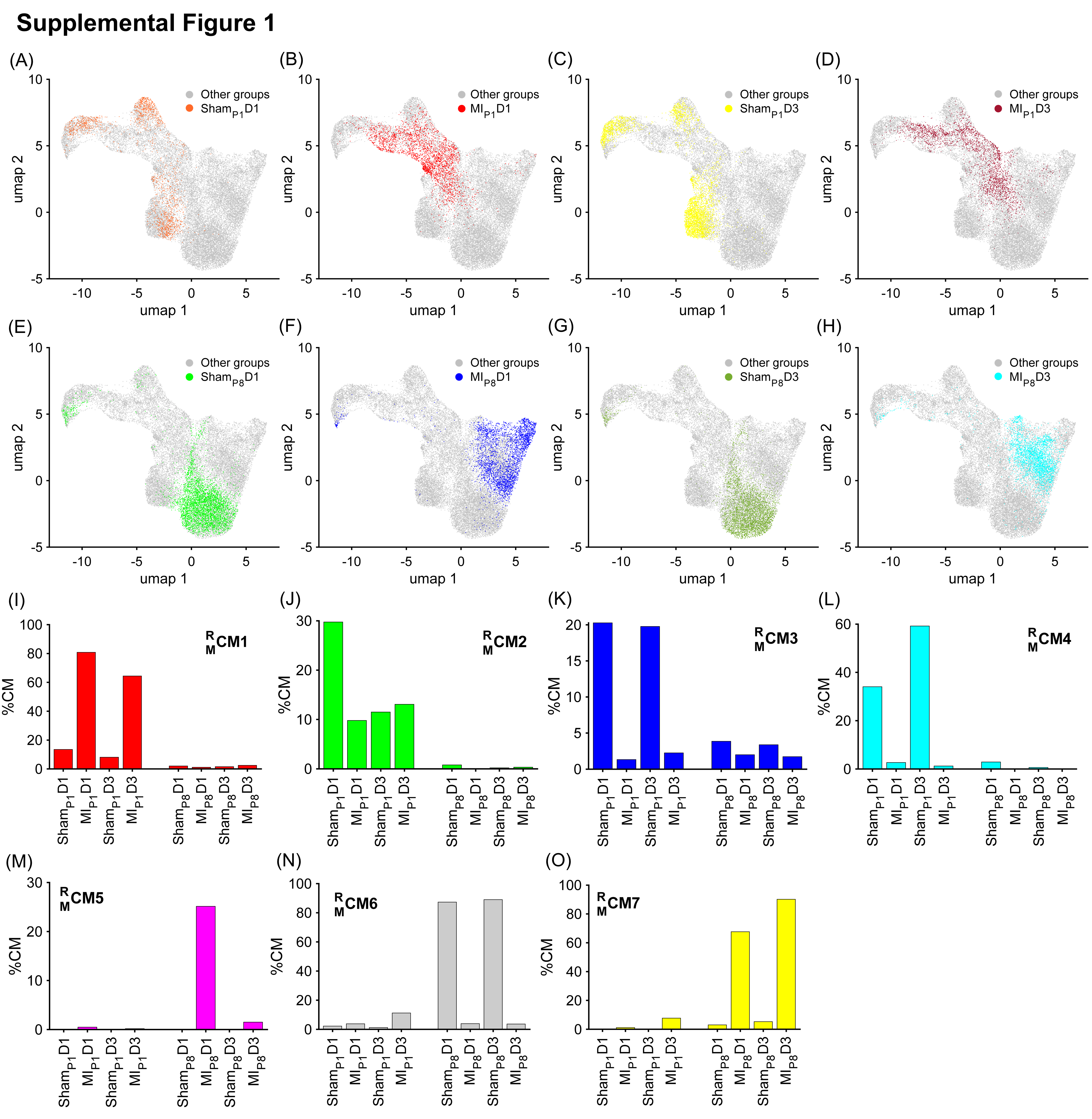

Supplement: Supplementary file 1 [file biomolecules-15-00310-s001.zip › Supplemental Figure S1.PNG]

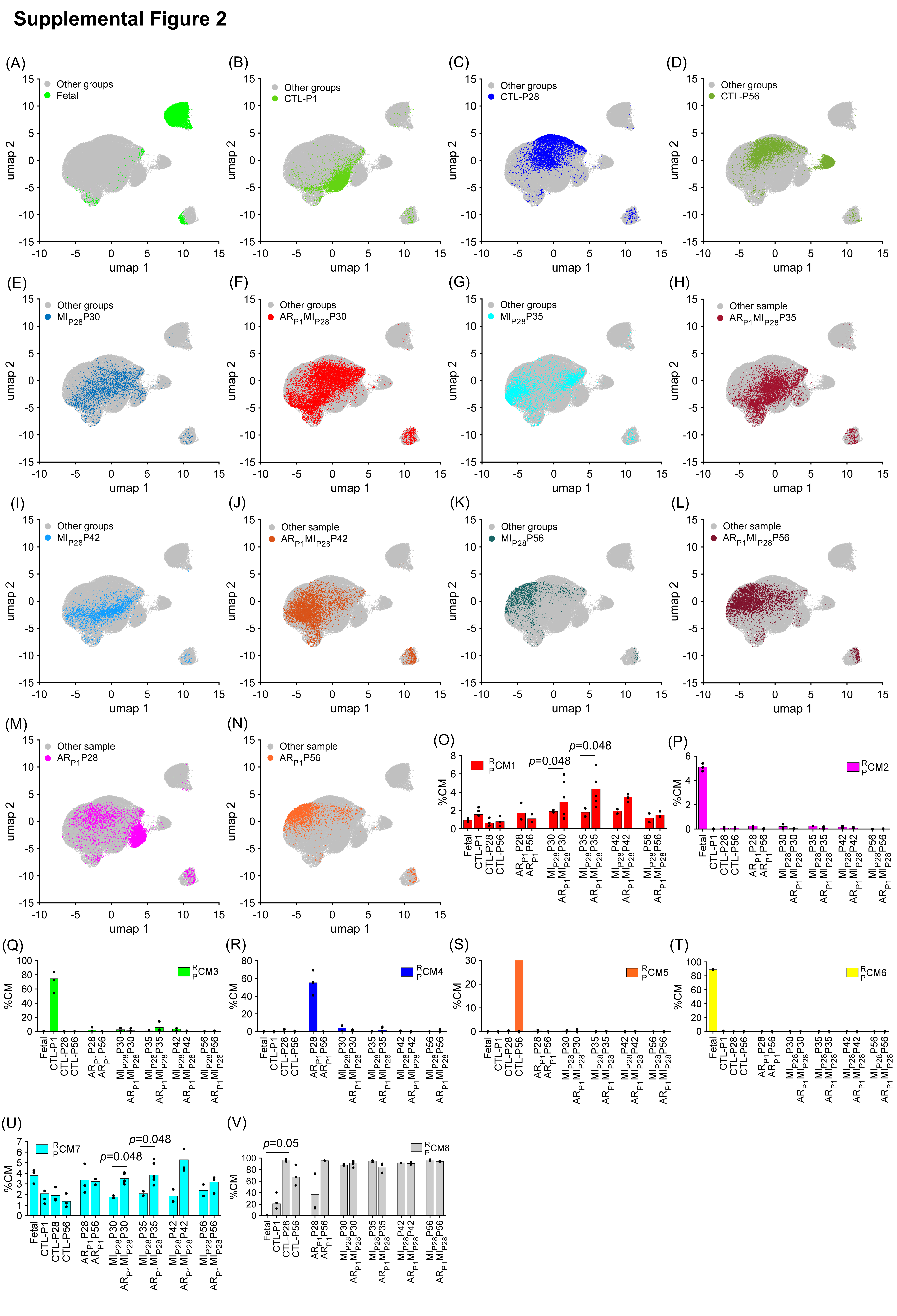

Supplement: Supplementary file 1 [file biomolecules-15-00310-s001.zip › Supplemental Figure S2.PNG]

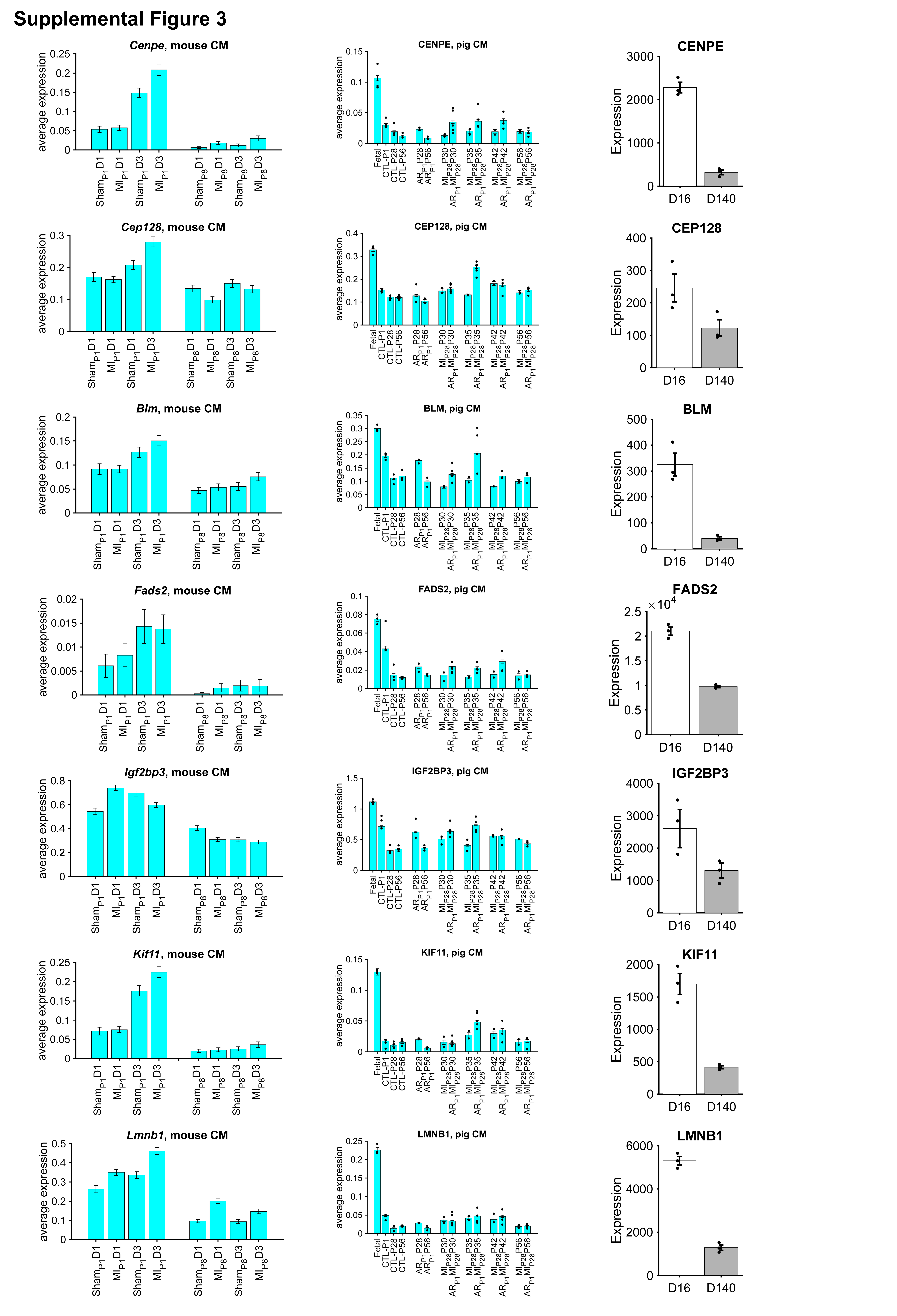

Supplement: Supplementary file 1 [file biomolecules-15-00310-s001.zip › Supplemental Figure S3-1.PNG]

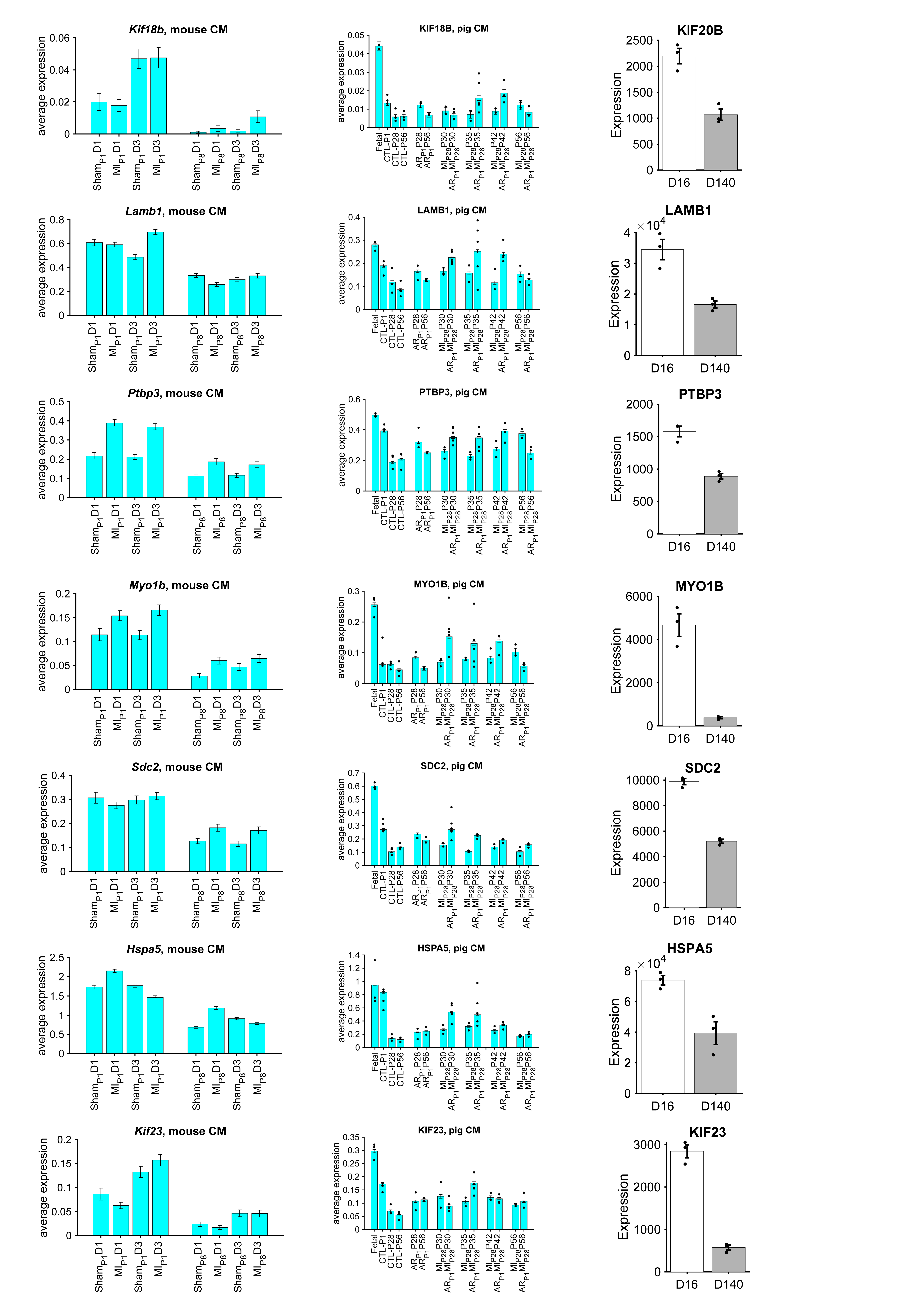

Supplement: Supplementary file 1 [file biomolecules-15-00310-s001.zip › Supplemental Figure S3-2.PNG]

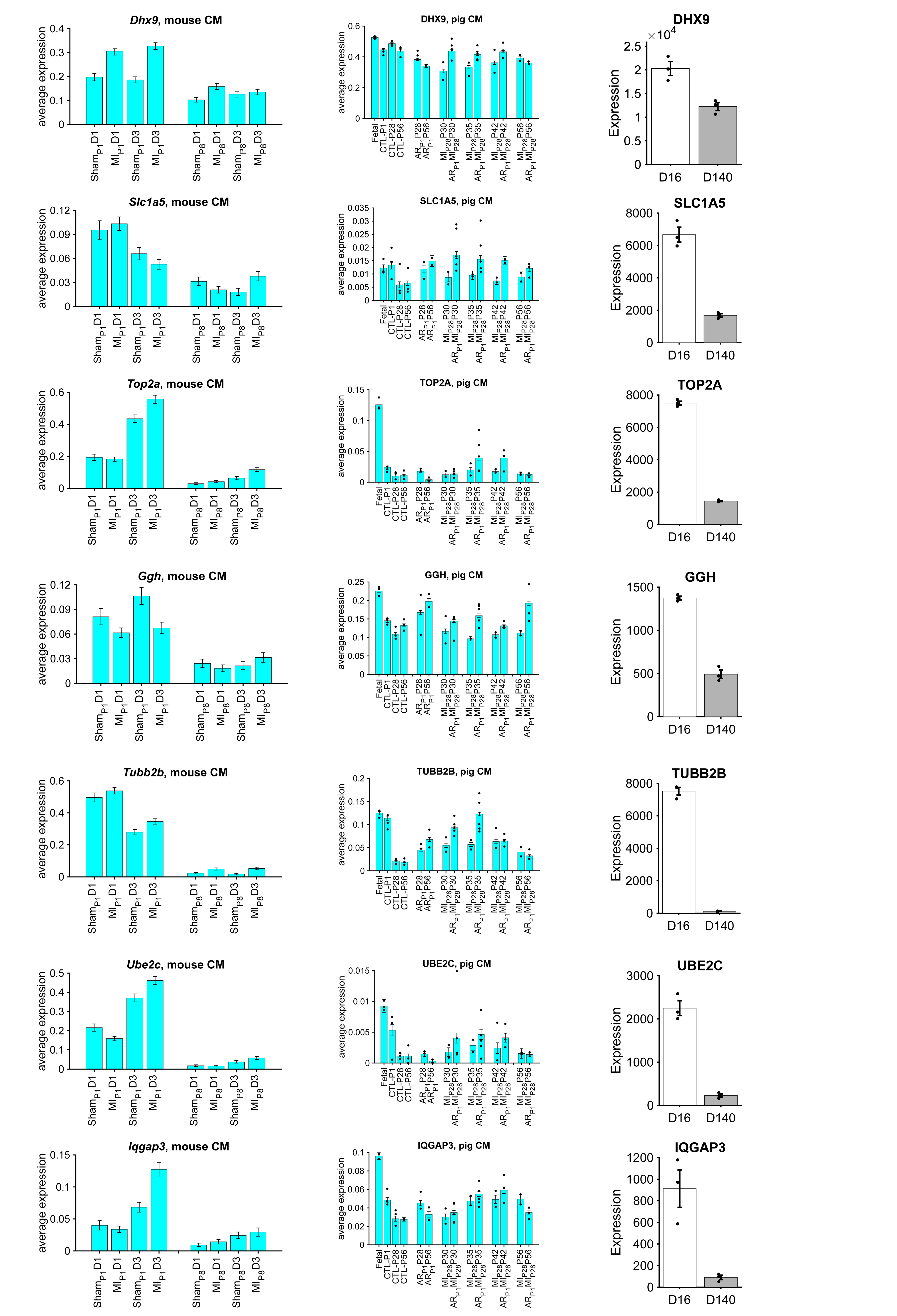

Supplement: Supplementary file 1 [file biomolecules-15-00310-s001.zip › Supplemental Figure S3-3.PNG]

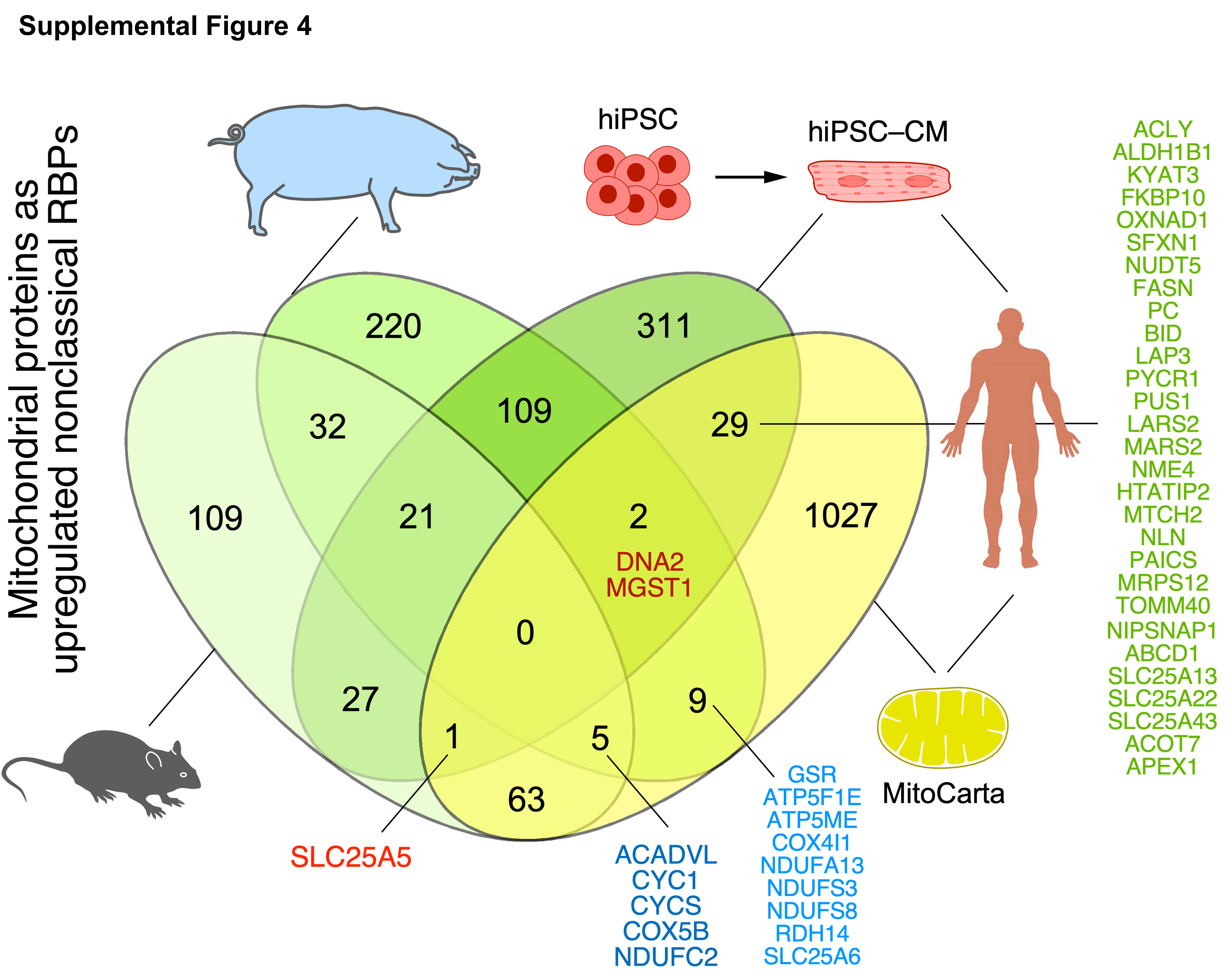

Supplement: Supplementary file 1 [file biomolecules-15-00310-s001.zip › Supplemental Figure S4.PNG]
